# Isolation of an ES-Derived Cardiovascular Multipotent Cell Population Based on VE-Cadherin Promoter Activity

**DOI:** 10.1155/2016/8305624

**Published:** 2016-12-22

**Authors:** Violetta A. Maltabe, Eleonora Barka, Marianthi Kontonika, Dimitra Florou, Maria Kouvara-Pritsouli, Maria Roumpi, Simeon Agathopoulos, Theofilos M. Kolettis, Panos Kouklis

**Affiliations:** ^1^Laboratory of Biology, Medical School, University of Ioannina, Ioannina, Greece; ^2^Department of Biomedical Research, Institute of Molecular Biology & Biotechnology, Foundation of Research and Technology-Hellas, University Campus, 45110 Ioannina, Greece; ^3^Ceramics and Composites Laboratory Department of Materials Science and Engineering, University of Ioannina, Ioannina, Greece; ^4^Department of Cardiology, University of Ioannina Medical School, Ioannina, Greece; ^5^Cardiovascular Research Institute, Ioannina, Greece

## Abstract

Embryonic Stem (ES) or induced Pluripotent Stem (iPS) cells are important sources for cardiomyocyte generation, targeted for regenerative therapies. Several in vitro protocols are currently utilized for their differentiation, but the value of cell-based approaches remains unclear. Here, we characterized a cardiovascular progenitor population derived during ES differentiation, after selection based on VE-cadherin promoter (Pvec) activity. ESCs were genetically modified with an episomal vector, allowing the expression of puromycin resistance gene, under Pvec activity. Puromycin-surviving cells displayed cardiac and endothelial progenitor cells characteristics. Expansion and self-renewal of this cardiac and endothelial dual-progenitor population (CEDP) were achieved by Wnt/*β*-catenin pathway activation. CEDPs express early cardiac developmental stage-specific markers but not markers of differentiated cardiomyocytes. Similarly, CEDPs express endothelial markers. However, CEDPs can undergo differentiation predominantly to cTnT^+^ (~47%) and VE-cadherin^+^ (~28%) cells. Transplantation of CEDPs in the left heart ventricle of adult rats showed that CEDPs-derived cells survive and differentiate in vivo for at least 14 days after transplantation. A novel, dual-progenitor population was isolated during ESCs differentiation, based on Pvec activity. This lineage can self-renew, permitting its maintenance as a source of cardiovascular progenitor cells and constitutes a useful source for regenerative approaches.

## 1. Introduction

Regeneration of ventricular myocardium has been at the center of research efforts during the past decade. Embryonic Stem (ES) or induced Pluripotent Stem (iPS) cells are important cellular sources towards this aim. Reproduction of the sequential stages of cardiac differentiation has been established during pluripotent ESCs differentiation under appropriate conditions in vitro [1]. In general ES-derived cells are isolated either as terminally differentiated cardiomyocytes [2–4] or as cardiovascular progenitor populations left to further differentiate after transplantation in vivo [5, 6]. The therapeutic potential of such isolated cardiogenic progenitors, even limited, has been reported in numerous studies [7–9]. Despite the advent of various protocols utilized for cardiomyocyte generation, the value of cell-based approaches for cardiac regeneration remains unclear. Specifically, the homing properties, survival, proliferation, and maturation of transplanted cells in the environment of myocardium are challenges that remain to be addressed [10–12].

Isolation and expansion of novel multipotent cardiovascular progenitors with limited differentiation potential could present a valuable tool towards this goal. Genetic-based ESCs differentiation systems take advantage of developmental stage specific activity of promoters for selection of cell populations. VE-cadherin is an adhesion molecule that contributes to adherens junctions formation between endothelial cells. VE-cadherin promoter (Pvec) has been previously characterized as endothelial specific in vivo and in vitro [13–16]. However, its transient activation was also detected in hemopoietic progenitor populations called “hemogenic endothelium” [17–19]. In our laboratory, we have previously analyzed Pvec activation during ESCs differentiation and found evidence of such activation in a subset of early Isl1^+^ cardiovascular progenitors (Maltabe et al., submitted). Isl1 belongs to a group of lineage-specific transcriptions factors expressed in early cardiogenesis [20]. Particularly, Isl1^+^ cells have been characterized as multipotent cardiovascular progenitors, because they differentiate further to cardiomyocytes, endothelial, endocardial, and smooth muscle cells [21].

In the present study, we aimed to generate and isolate a novel cardiovascular progenitor population derived from ESCs, based on genetic selection strategy. Towards this aim we used Pvec activity to drive an antibiotic resistance gene expression during ESCs differentiation and we provide evidence that a cardiovascular progenitor population can be isolated by this strategy. We further show that this population has the capacity to self-renew and differentiate to cardiac and endothelial cells under specific cell culture conditions. Moreover, these cells survived and differentiated after direct intramyocardial transplantation in the left ventricle of adult rats.

## 2. Materials and Methods

### 2.1. Plasmids

#### 2.1.1. Pvec

An ~2.5 kb fragment containing mouse VE-cadherin promoter elements and the first nontranslated exon was derived by PCR using primers AGCAGAAACAAGGTCCTCTGGAAGAG (sense) and TCACTTACCTTGTCCGTGAGC (antisense) from a mouse BAC library as template, further subcloned in Topo-XL vector (Invitrogen).

#### 2.1.2. Ppvec-puro

The following subcloning steps were performed: Construct A, the chimeric gene and stuffer fragment of pPyCAGIP (an episomal vector, kind gift from Professor A. Smith, Wellcome Trust Centre for Stem Cell Research, University of Cambridge, UK), was inserted in the Topo-XL vector downstream of the mouse Pvec by SchI/EcoRI-blunt ligation. The SpeI/XhoI fragment from construct A was ligated to pPyCAGIP. Finally, a hygromycin resistance gene was inserted at NdeI blunt/SalI.

#### 2.1.3. Ppvec-puro-EGFP

EGFP coding sequence (from pEGFP-N) digested with Xho/NotI was ligated to the same sites of pPvec construct.

### 2.2. Cell Culture

E14T Embryonic Stem Cells (ESCs) were kindly provided by Professor A. Smith and Dr. I. Chambers (MRC Centre for Regenerative Medicine, Edinburgh, UK). They were propagated on gelatin (0.1% swine skin), in high glucose Glasgow modified Eagle's medium (Sigma) supplemented with LIF conditioned medium, 15% FBS (Biochrom), 1 mM Sodium Pyruvate (Invitrogen), 2 mM L-Glutamine (Invitrogen), 0.1 mM nonessential amino acids (Invitrogen), 0.05 mM *β*-mercaptoethanol (Sigma), 100 u/mL penicillin, and 0.1 mg/mL streptomycin (Invitrogen).

### 2.3. Stable ES Cell Lines

5 × 10^6^ ESCs were electroporated with 20 *μ*g of DNA in 600 *μ*L PBS at 200 V and 960 uF in a 0.4 cm cuvette using BTX-ECM600 electroporator (Harvard Apparatus). After 24 h and for the following 14 days, cells were selected with hygromycin (150 ug/mL to 120 ug/mL). Resistant clones were isolated and propagated individually.

#### 2.3.1. ESCs Differentiation

*(a) In Mass Culture*. 1 × 10^6^ ESCs were seeded on 100 mm bacteriological Petri dishes in differentiation medium DM1. DM1 is Iscove's modified Dulbecco's medium (IMDM), supplemented with 15% FBS, 2 mM L-Glutamine, 100 u/mL penicillin, 0.1 mg/mL streptomycin, 5 ng/mL human VEGF (ImmunoTools), 30 ng/mL human bFGF (ImmunoTools), and 5 *μ*g/mL Ascorbic Acid (Sigma). Briefly, ESCs were trypsinized and suspended in DM1 medium, and cells were cultured for three days in a 37°C humidified incubator with 5% CO^2^.

*(b) By “Hanging Drops.”* ESCs were seeded at 500 cells per 20 *μ*L drop in DM1 and cultured in hanging drops for 2 days. EBs formed were collected and plated on bacterial Petri dishes for further differentiation.

#### 2.3.2. Puromycin Selection during Differentiation

EBs were formed in mass culture for three days in culture medium DM1 that supports endothelial and cardiac differentiation. At day 3 EBs were dissociated by mild trypsin treatment and seeded on fibronectin coated tissue culture plates. For selection, puromycin (0.75 ug/mL to 1.5 ug/mL) was added at day 4.5 to day 8.

#### 2.3.3. Differentiation of CEDPs

CEDPs were trypsinized and suspended in DM2. DM2 is Iscove's modified Dulbecco's medium (IMDM), supplemented with 15% FBS, 2 mM L-Glutamine, 100 u/mL penicillin, 0.1 mg/mL streptomycin, 10 ng/mL human VEGF (ImmunoTools), 30 ng/mL human bFGF (ImmunoTools), and 5 *μ*g/mL Ascorbic Acid (Sigma). Spheres formed in low attachment plates were cultured in a 37°C humidified incubator with 5% CO^2^.

#### 2.3.4. Differentiation of CEDPs in Alginate (Pronova, Oslo, Norway)

CEDPs were resuspended at a density of 1 × 10^6^ cells/mL in 1.1% gelatinized alginate solution. This solution was added dropwise in 1 M CaCl_2_ pH7.4 through an insulin syringe and the encapsulated cells were washed in 0.9% NaCl, resuspended in DM2 medium. To increase viability of CEDPs, they were mixed with gelatinized alginate solution and were injected in the left ventricle of rats in vivo. Under these conditions, alginate solution is known to cross-link with endogenous calcium ions, quickly forming hydrogel [22].

#### 2.3.5. In Vivo Study Population and Ethics

The in vivo experiments were conducted on 15 Wistar rats (all male, 17–20 weeks of age, weighing 280–320 g). The animals were housed in plexiglas-chambers in groups of two or four, with free access to water and standard rodent pellet-diet. The housing facilities at the University of Ioannina adhere to international guides and offer stable conditions, in terms of temperature (20–22°C), humidity (60–70%), and light-to-dark cycles (12 : 12 h). The experimental procedures followed the guiding principles of the Declaration of Helsinki, regarding ethical conduct of animal research, and conformed to European legislation (European Union directive for the protection of animals used for scientific purposes 609/1986, revised in 2010/63/EU). The study protocol was approved by the Department of Agricultural Economy and Veterinary Medicine, Ioannina, Prefecture of Epirus, approval number 6003, 19/04/2013.

### 2.4. Implantation Protocol

Following anesthesia induction with isoflurane-inhalation via mask, the rats were intubated and mechanically ventilated using a rodent apparatus (model 7025, Ugo Basile); anesthesia was maintained with a mixture of oxygen and 2.5% sevoflurane.

Via left lateral thoracotomy, the heart was exposed, and the pericardium was removed; implantation was performed by intramyocardial injections, as described previously [23]. In brief, the heart was exteriorized and slight traction was applied via a 6-0 suture, passed through the apex, thereby facilitating manipulations and providing support during injections. A total of 0.2 mL of normal saline (*n* = 5) or alginate-hydrogel with CEDPs (*n* = 10) was administered by six intramyocardial injections in the anterolateral LV wall, as in previous experiments [24] occasional bleeding stopped after light pressure was applied locally. The incision was closed in three layers and pneumothorax was evacuated. For analgesia, a single intraperitoneal injection of an opioid-analgesic (buprenorphine, 0.05 mg/kg) was administered postoperatively.

### 2.5. Immunosuppression Protocol

To prevent allograft rejection, low-dose immunosuppression was administered, as outlined previously [25, 26]. Specifically, cyclosporine (10 mg/kg) was administered orally by gavage, starting from the day prior to implantation, until the end of the experiment.

Heart specimens were harvested three (*n* = 3), seven (*n* = 3), and 14 days (*n* = 4) after implantation. The animals were anesthetized (as described above), and the site of previous thoracotomy was reopened. The aorta, pulmonary artery, and superior and inferior vena cava were clamped; the heart was excised and quickly immersed in normal saline. Subsequently, hearts were processed for immunocytochemistry or RNA isolation.

### 2.6. Immunocytochemistry

EBs and spheres were allowed to attach on gelatinized glass coverslips for 2 days before staining. Cells were fixed in 4% formaldehyde for 10 min at RT. Subsequently, they were incubated with 3% BSA containing 0.2% Triton-X100 for 30 min and primary antibody labeling was performed at 4°C O/N, followed by incubation with the secondary antibody for 1 h. For microscopy, rat hearts were fixed in 4% formaldehyde for 2 h and then 30% sucrose over-night and then embedded in OCT, sectioned and stained using standard protocols. In brief, frozen tissue sections were permeabilized with 100% ice-cold methanol for 10 minutes at −20°C and rinsed in PBS for 5 minutes. Antibody labeling was carried out as above, with the exception that primary antibody was diluted in 0.2% fish skin gelatin and labeling was performed for 1 h at room temperature.

#### 2.6.1. Antibodies

For immunocytochemistry, the following antisera were used: rat monoclonals against VE-cadherin (11D4.1, BD Biosciences), PECAM-1 (MEC 13.3, Santa Cruz), and E-Cadherin (DECMA-1, Santa Cruz), mouse monoclonals against cardiac Troponin T (CT3, Iowa Hybridoma Bank), Isl1 (39.4D5, Iowa Hybridoma Bank), Oct3/4 (C-10, Santa Cruz), SMA (Neomarkers), N-cadherin (clone 3B9, Invitrogen), MyHC (MF20, Iowa Hybridoma Bank), and a-actinin (Clone BM-75.2, Sigma), goat polyclonals against GATA4 (C-20, Santa Cruz) and Isl1 (GT15051-100, Acris Antibodies), rabbit monoclonals against MEF2c (D80C1) and VEGF receptor 2 (Flk1) (55B11) from Cell Signaling, and rabbit polyclonals against EGFP (kindly provided from Dr. Charalambia Boleti, Pasteur Institute, Athens), Desmoplakin 1/2 [27], and DSC2 (DSC2, RDI Research Diagnostics, Inc.).

### 2.7. Confocal Microscopy

Confocal images were taken in a Leica confocal microscope (LCS SP5) using the LAS AF Lite software. Pictures were further manipulated with Fiji (NIH Image) and/or Adobe Photoshop (Adobe) software.

### 2.8. RNA Isolation, rt-PCR, and Quantitative rt-PCR

RNA was isolated using TRIzol reagent according to manufacturer's protocol (Invitrogen). To synthesize cDNA 1 *μ*g of purified RNA was used in 20 *μ*L reaction, using PrimeScript™ RT reagent Kit with gDNA Eraser (Takara). Quantitative real time PCR analysis was performed with one-twelfth or one-sixth of the cDNA reaction as template, using KAPA SYBR® FAST qPCR Kit Master Mix (Kappa) in Bio-Rad CFX96 for 45 cycles. All samples were analyzed in triplicates. All values were normalized with respect to GAPDH and *β*-actin expression levels, translated to relative values. Analysis was performed by qBase plus software (Biogazelle). Primer sequences are shown in Supplementary Table S1 in Supplementary Material available online at http://dx.doi.org/10.1155/2016/8305624.

### 2.9. FACS Analysis

Cells during selection from differentiating cultures were trypsinized using 0.25% trypsin-EDTA and EGFP expression was analyzed by FACS (Partec CyFlow Space). Data obtained were analyzed with FCS Express 4 software (Flow Research Edition).

### 2.10. Cell Growth Analysis

1 × 10^6^ Pvec^+^ cells were seeded in a 30 mm tissue culture plate. Upon reaching approximately 80% confluence, the cells were treated with 0.05% trypsin-EDTA solution (Gibco) for detachment and counted before reseeded in a new tissue culture plate and cultured until the next passage. This process was repeated up to ten passages. The doubling time was calculated using Doubling Time Computing [28].

### 2.11. Statistical Analysis

Statistical Analysis was performed with GraphPad Prism 5 Software. Data represent the mean ± SD from three independent experiments. The statistical significance of difference was determined by one-way ANOVA followed by Tukey's Multiple Comparison Test. Probability values *P* < 0.05 were considered significant. For calculating the total cell numbers in heart tissue (Figure 7(e)) the computer algebra system Maple 18 and package Curve Fitting were used.

## 3. Results

### 3.1. Isolation of Cells with Cardiac and Endothelial Phenotypes during ES Cells Differentiation by Selection Based on Pvec Activity

**Figure 1:**
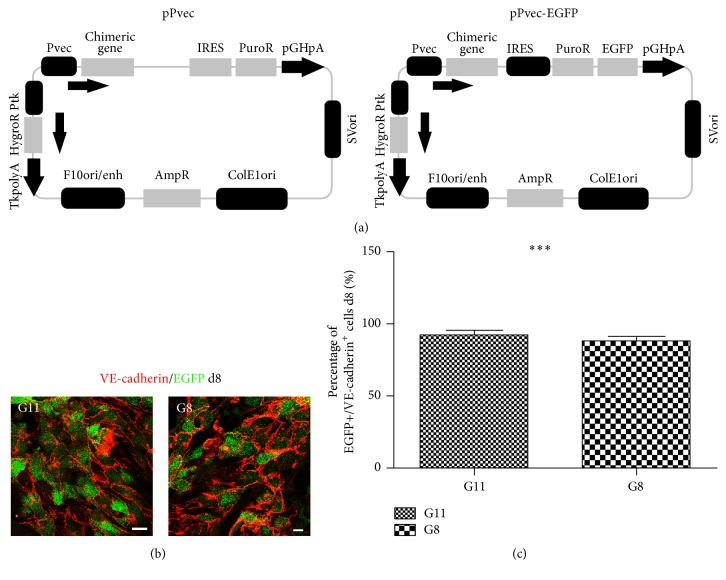
Generation and characterization of genetically modified ES clones expressing puromycin resistance gene under Pvec. (a) Schematic representation of episomal constructs pPvec and pPvec-EGFP, used for generation of ES clones. (b) The majority of VE-cadherin^+^ cells express EGFP in EBs derived from clones G11 and G8 at d8. (c) Statistical analysis of the percentage of VE-cadherin^+^ cells coexpressing EGFP at d8 from three independent experiments. For each experiment ~600 VE-cadherin^+^ cells were counted. ^*∗∗∗*^ *P* < 0.001. Scale bar: 20 *μ*m.

In order to isolate ES-derived Pvec^+^ cells we genetically modified E14T ESCs. E14T cells were electroporated with an episomal plasmid containing the genes for puromycin resistance under Pvec and hygromycin resistance under Thymidine Kinase promoter (Figure 1(a)). Hygromycin resistant clones A11 and A12 (Pvec-ESCs) were isolated and expanded. In similar experiments, EGFP-expressing clones G8 and G11 under Pvec were also isolated (Pvec-EGFP ESCs) (Figure 1(a)). Clones were positive for pluripotency markers Oct4, Sox2, and E-cadherin and their differentiation properties were examined (Supplementary Figure S1). Tissue-specific Pvec activity was assessed during in vitro differentiation of G8 and G11 by EGFP expression (Figure 1(b)). EBs formed at d8 were double-stained for VE-cadherin and EGFP and the percentage of double-positive cells was calculated. More than 90% of EGFP^+^ cells were also VE-cadherin^+^, an indication of high promoter specificity (Figure 1(c)). VE-cadherin was found predominantly in the plasma membrane, as well as in the cytoplasm, forming nascent adherens junctions at this early developmental stage (Figure 1(b)).

**Figure 2:**
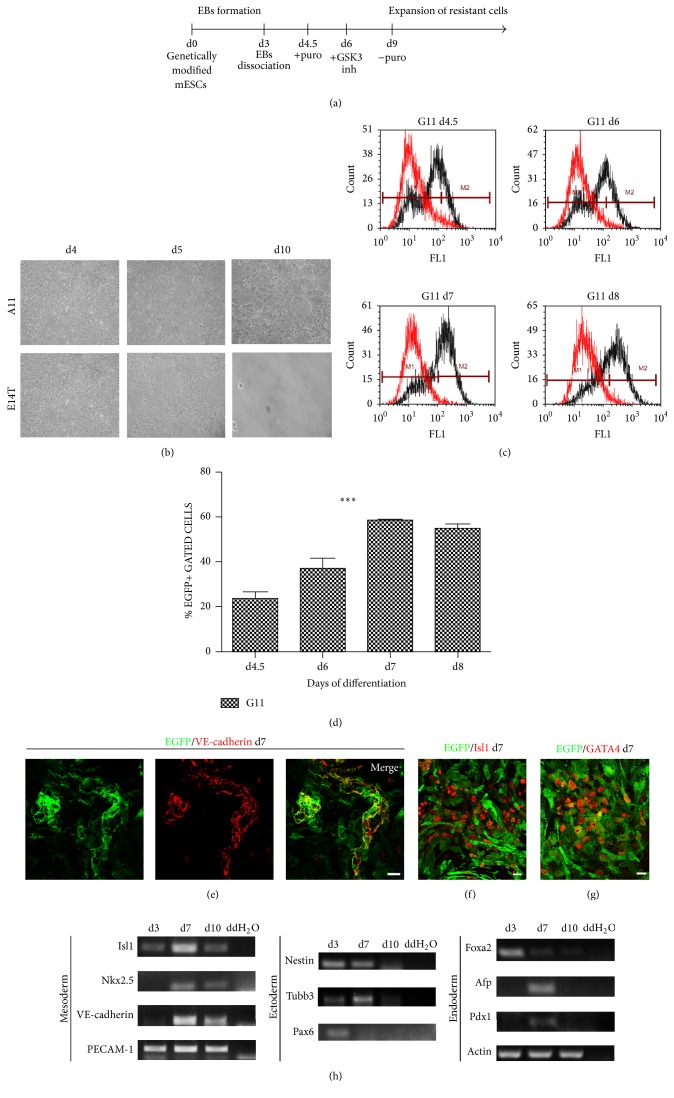
Selection of cells with Pvec activity during differentiation. (a) Schematic representation of selection strategy during differentiation of genetically modified ESCs to isolate Pvec^+^ cells. (b) Optical microscope images of clone A11- and E14T-derived cells, during selection at d4 (before the addition of puromycin), d5, and d10 (selected cells). (c) Quantification of EGFP^+^ cells during clone G11 differentiation/selection at d4.5, d6, d7, and d8 by FACS analysis (representative results are shown). (d) Analysis of FACS data from three independent experiments. ^*∗∗∗*^ *P* < 0.001. (e–g) G11-derived EGFP^+^ selected cells at d7 coexpress VE-cadherin, Isl1, and GATA4. Note that EGFP was detected at low Isl1 and GATA4 expressors. (h) Expression of mesodermal, endodermal, and neuroectodermal markers during selection of clone A11 at d3, d7, and d10 by RT-PCR analysis.

Next, we analyzed the surviving products of Pvec- and Pvec-EGFP ESCs during differentiation in puromycin selection DM1 medium (Figure 2(a)) (see Section 2). Pvec- and Pvec-EGFP-ESCs resistant cells were observed at d10, in contrast to wtESCs-derived cells that were eliminated (Figure 2(b)). FACS analysis for EGFP^+^ cells showed that they were 26% of the total cells at d4.5 and increased to 40% at d6 and 60% at d7 and d8 (Figures 2(c) and 2(d)).

Surviving cells were further analyzed for cardiac and endothelial specific markers expression by immunofluorescence at d7. They expressed endothelial VE-cadherin and PECAM-1 (Figures 2(e) and 2(h)), as well as cardiac developmental stage-specific markers Isl1, GATA4, and Nkx2.5 (Figures 2(f), 2(g), and 2(h)). Interestingly, VE-cadherin-mediated adherens junctions were compromised in the presence of puromycin. No endodermal or neuroectodermal specific markers were detected by RT-PCR at d3, d7, and d10 (Figure 2(h)). As control, we performed differentiation and selection experiments of clone A11 under the same conditions with hygromycin instead of puromycin. Since the episomal vector Pvec contains hygromycin resistance under the ubiquitous Thymidine Kinase (TK) promoter (Figure 1(a)), A11 cells survived and differentiated efficiently to neuroectodermal, endodermal, and mesodermal lineages (Supplementary Figure S2).

### 3.2. Activation of Wnt/*β*-Catenin Pathway Induces Propagation and Self-Renewal of Selected Pvec^+^ Cells

**Figure 3:**
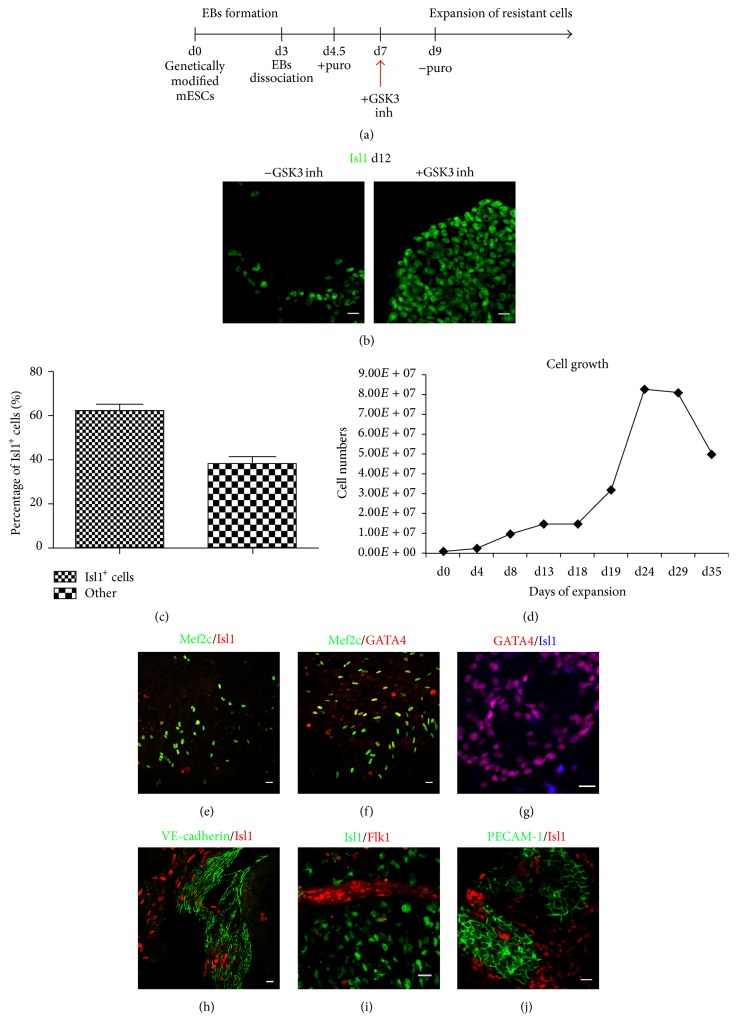
Expansion of Pvec^+^ cells. (a-b) Schematic representation of expansion strategy and propagation of Pvec^+^ cells. Addition of GSK3 inhibitor (SB-216763) induces propagation of Pvec^+^ cells expressing Isl1 at d12. (c) Pvec^+^/Isl1^+^ cells percentage in SB-216763 from three independent experiments. For each experiment ~1000 cells were counted. (d) Pvec^+^cell growth curve. (e–j) Propagated cells consist of cell populations expressing markers of cardiac (Isl1, Mef2c, and GATA4) and/or endothelial (Flk-1, PECAM-1, and VE-cadherin) progenitors.

We observed that VE-cadherin^+^ and Isl1^+^ surviving cells could not grow further after d14 in DM1 medium. In an attempt to expand Isl1^+^ cells, we induced Wnt/*β*-catenin, a signaling pathway known to support self-renewal of Isl1^+^ cardiac cells (Figure 3(a)). When SB-216763 [29], a GSK3 inhibitor, was added at days 5–8, significant expansion of Isl1^+^ cells was observed at d12 (Figure 3(b)).

The percentage of Isl1^+^ cells calculated by cell-counting was found to exceed 60% of total cells (Figure 3(c)). These properties were maintained for at least eight passages up to 30 days and multiple freeze and thaw cycles. Afterwards their growth declined substantially. The doubling time of Pvec^+^ cells in the presence of SB-216763 calculated after growth curve generation between day 1 and day 28 was ~4.5 days (Figure 3(d)).

**Figure 4:**
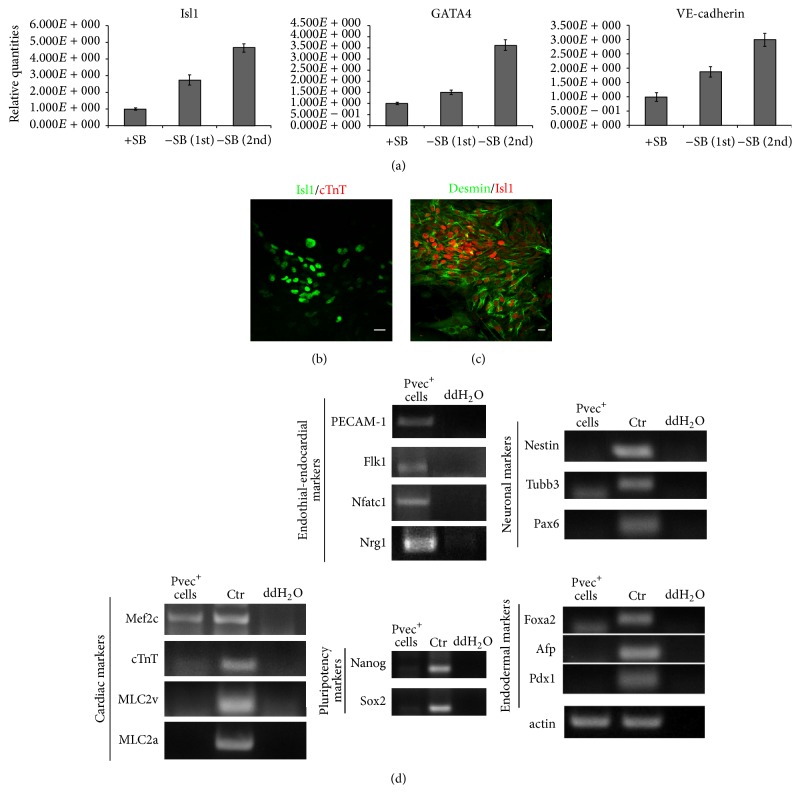
Characterization of Pvec^+^ cells. (a) Isl1, GATA4, and VE-cadherin mRNA levels quantification in Pvec^+^ cells in the presence or absence of SB-216763 by real time qPCR. Results in -SB first and -SB second referred to two independent selection/differentiation experiments. (b-c) Isl1^+^ cardiac progenitors express desmin but not cTnT, a marker of differentiated cardiomyocytes. (d) RT-PCR analysis of expanded Pvec^+^ cells showed expression of cardiac, endothelial, and endocardial but not neuroectodermal, endodermal, or pluripotency markers. As positive controls (ctr) mRNA from E14T ESCs differentiation was used. Note the lack of cTnT, MLC2v, and MLC2a expression in expanded Pvec^+^ cells. Scale bar: 20 *μ*m.

Cells were examined for self-renewal by cardiomyocyte differentiation stage-specific markers expression. We analyzed for cardiac progenitor markers GATA4 and Mef2c expression and found that Mef2c, a marker of the AHF (anterior heart field), is coexpressed with Isl1 and GATA4 in the majority of cells (Figures 3(e)–3(g)). During SB-216763 induced propagation it became evident that endothelial cells also survived, proliferated efficiently, and formed extensive VE-cadherin-mediated adherens junctions and PECAM-1 junctions, as shown by immunofluorescence staining (Figures 3(h)–3(j)). Isl1, VE-cadherin, and GATA4 expression levels were quantified and compared between Pvec^+^ cells expanded in SB-216763 and Pvec^+^ cells after selection by qPCR. In SB-treated cultures Isl1 was found upregulated 2.7- and 4.7-folds, GATA4 was upregulated 1.5- and 3.6-folds, and VE-cadherin was upregulated 1.8- and 3-folds in two biological independent selection/expansion experiments (Figure 4(a)).

Further cardiac differentiation was inhibited as they were negative for MLC2v, MLC2a, and cTnT, although they coexpressed Desmin and Isl1 (Figures 4(b), 4(c), and 4(d)). This is consistent with the role of Wnt/*β*-catenin signaling pathway known to induce proliferation and inhibit Isl1^+^ cells differentiation.

Endothelial cells also did not differentiate further, as vWF and CD39 markers of mature endothelium were not expressed (Supplementary Figure S3). Interestingly, expression of Nfatc1 and Nrg1 was detected, implying the presence of endocardial cells (Figure 4(d)). In control experiments pluripotency markers Nanog and Sox2 were not expressed in these cells, shown by RT-PCR analysis (Figure 4(d)).

Thus, under the protocol described above propagation of a cardiac/endothelial dual-progenitor population (called CEDPs) was achieved.

### 3.3. CEDPs Differentiation to Cardiac and Endothelial Cells In Vitro

**Figure 5:**
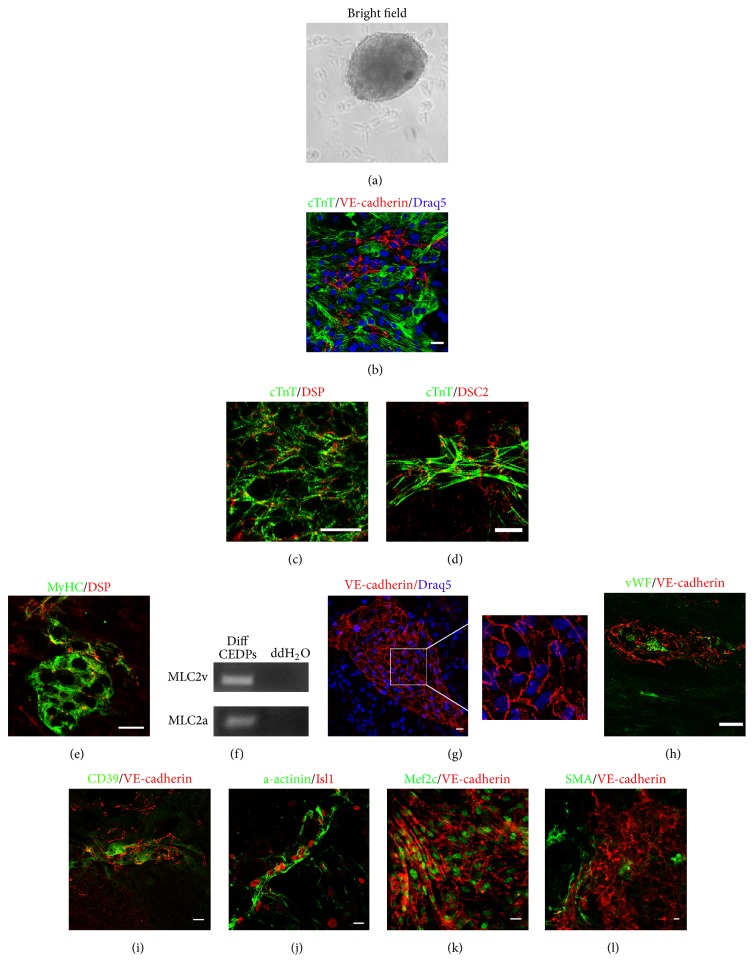
CEDPs differentiation potential. (a) Optical microscope image of a sphere formed in the absence of SB-216763 after 5 days of CEDPs differentiation. (b) cTnT^+^ cells formation after immunostaining with anti-cTnT and anti-VE-cadherin. (c–e) Formation of intercalated disk structures in beating spheres after 10 days of CEDPs differentiation, shown by double-IF-staining with anti-cTnT/anti-Desmoplakin (DSP), anti-cTnT/anti-Desmocollin-2 (DSG2), and anti-MyHC/anti-Desmoplakin. Note the striations of the sarcomeric cTnT staining and the punctate desmosomal staining in areas connecting adjacent cardiomyocytes. (f) Expression of MLC2v and MLC2a after 10 days of CEDPs differentiation by RT-PCR analysis. (g) VE-cadherin^+^ cells in CEDPs-derived differentiated cells. Note extensive adherens junctions formation between endothelial cells. Magnification corresponds to marked area. (h-i) Expression of vWF and CD39 in VE-cadherin^+^ endothelial cells after 10 days of CEDPs differentiation. (j-k) Expression of Isl1 and Mef2c progenitor markers in a-actinin^+^ and VE-cadherin^+^ cells, respectively, at day 10 of CEDPs differentiation. (l) Expression of SMA during CEDPs differentiation. Draq5 counterstained DNA (b, g). Scale bar: 20 *μ*m.

We examined next the potential of CEDPs to differentiate further towards cardiac and endothelial cell types. For this purpose, CEDPs were cultivated in differentiation medium DM2 in the presence or absence of SB-216763 in low adhesion plates. SB-216763 inhibited differentiation, evident by small spheres formation with no beating activity. In contrast, in the absence of SB-216763 approximately 3-4-fold larger spheres with beating phenotype were formed after 10 days (Figure 5(a) and Supplementary Figure S4). Spheres contained extensive areas of cTnT^+^ or MyHC^+^ cells coexpressing adhesion molecules Desmoplakin and Desmocollin 2, an indication of intercalated disk structures formation between cardiomyocytes (Figures 5(c), 5(d), and 5(e))). MLC2a and MLC2v markers of differentiated cardiomyocytes were also detected in such cultures by RT-PCR analysis, in contrast to CEDPs (Figures 5(f) and 4(d)). Induction of endothelial markers vWF and CD39 and VE-cadherin^+^ cobble-stone structures formation observed during differentiation of CEDPs indicate maturation of endothelial cells (Figures 5(g)–5(i)). Interestingly, cells expressing progenitor markers Isl1 and Mef2c could be detected in cardiac and endothelial cells, respectively, during differentiation (Figures 5(j) and 5(k)). In addition, SMA^+^ cells could also be observed (Figure 5(l)).

**Figure 6:**
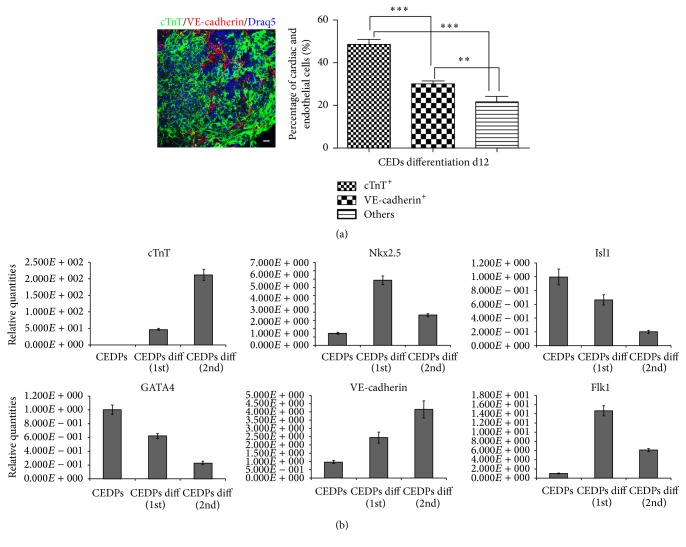
Characterization of CEDPs-derived products. (a) Statistical analysis of cTnT^+^ and VE-cadherin^+^ cells in CEDPs-derived differentiated cells at d12. The total number of cells counted was 10417 in three independent experiments. ^*∗∗∗*^ *P* < 0.001 and ^*∗∗*^ *P* < 0.01. Cells from random fields were photographed (as in representative image left) and counted by Fiji cell-counter. Scale bar: 20 *μ*m. (b) cTnT, Nkx2.5, Isl1, GATA4, VE-cadherin, and Flk1 mRNA levels quantification in CEDPs-derived differentiated cells at d10 by real time qPCR. Results in CEDPs differentiation first and CEDPs differentiation second referred to two independent differentiation experiments.

Cardiac and endothelial cells derived during CEDPs differentiation at day 12 were quantified by counting cTnT^+^ and VE-cadherin^+^ cells in three independent experiments (total of 10417 cells). We found that 47% of cells were cTnT^+^, 28% were VE-cadherin^+^, and the remaining nonendothelial, noncardiac cells (approximately 25%) were positive for smooth muscle actin or vimentin (Figure 6(a) and Supplementary Figure S5), possibly representing a cell population related to cardiac mesenchyme. cTnT, VE-cadherin, Isl1, GATA4, Nkx2.5, and Flk1 expression levels were quantified and compared in CEDPs and in CEDP-derived differentiation cells at day 12 by qPCR. We observed upregulation of cTnT, Nkx2.5, VE-cadherin, and Flk1 and downregulation of Isl1 and GATA4 (Figure 6(b)). These results show that CEDPs can differentiate further to cardiomyocytes.

In control experiments differentiation towards endoderm and neuroectoderm was not observed in spheres, since lineage-specific markers (FoxA2, AFP, and Pdx1 for endoderm and Tub-b3, nestin, and Pax6 for neuroectoderm) were not detected by RT-PCR analysis (Supplementary Figure S6).

CEDPs differentiation properties imply that they could potentially represent a progenitor population useful for cardiac regeneration. Therefore, we examined their differentiation efficiency in alginate, a hydrogel biomaterial commonly used as a scaffold in transplantation experiments [30]. CEDPs were encapsulated in alginate and cultured in DM2 medium as above (Supplementary Figure S7). Spheres were formed and remained up to 10 days in hydrogel when the biodegradable material started to disintegrate. We observed that CEDPs differentiated in alginate in a similar manner compared to liquid DM2 media, evident by cardiac beating, observed after 12 days.

### 3.4. Survival and Differentiation of CEDPs after Transplantation

**Figure 7:**
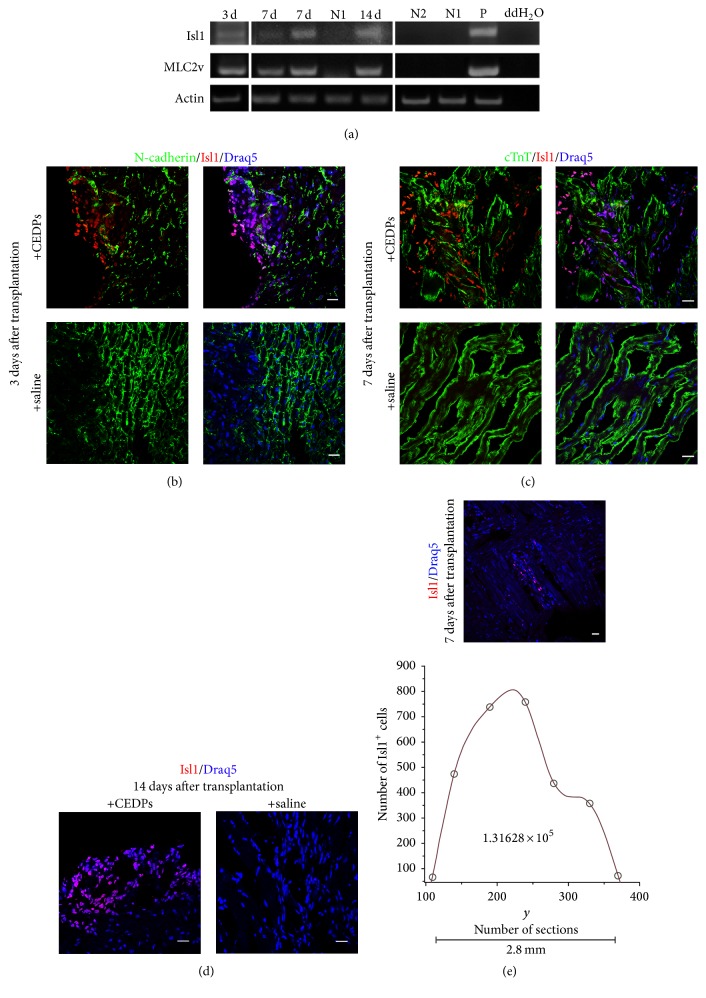
Transplantation of CEDPs in adult rats' heart. (a) Expression of Isl1 and MLC2v after 3, 7, and 14 days of transplantation by RT-PCR analysis. mRNA from saline-injected immunosuppressed (N1) or untreated (N2) LV of adult rats was used as negative controls and mRNA from E12 mouse embryos (P) as positive control. (b–d) Clusters of Isl1^+^ cells derived from CEDPs were detected in frozen sections of LVs isolated 3, 7, and 14 days after transplantation. Sections were stained for DNA with Draq5 and with anti-N-cadherin (b) and anti-cTnT (c). Sections from isolated LVs of saline-injected immunosuppressed rats were used as control for each time-point. (e) All Isl1^+^ cells from two nonsequential sections were photographed (as in representative upper image) and counted with Fiji cell-counter. Draq5 used for DNA staining. The total Isl1^+^ cell number found in 2.8 mm tissue was calculated after spline interpolation. Scale bar: 20 *μ*m.

Survival and differentiation of CEDPs were examined in vivo after transplantation in the left ventricle (LV) of immunosuppressed rats. Distinction between CEDPs-derived and endogenous cardiac cells was based on cardiac progenitor marker Isl1 present only in CEDPs and their progeny but not in the LV of the adult heart. For this purpose, the LV areas from CEDPs or saline recipient rats were dissected and examined after 3, 7, and 14 days by immunocytochemical and RT-PCR analysis for survival and differentiation of CEDPs. Immunostaining of frozen sections demonstrated in all cases that Isl1^+^ cell populations appeared specifically in CEDPs- but not in saline-injected animals. RT-PCR analysis also showed that Isl1^+^ expression could not be detected in this heart portion (Figure 7(a)). Expression of pluripotency markers Oct4 and Nanog was not detected in transplanted animals examined (Supplementary Figure S8).

Differentiation of CEDPs was monitored by RT-PCR for MLC2v, a differentiation marker not expressed in CEDPs using a mouse-specific primer set (Figure 4(a) and Supplementary Figure S9).

CEDP-derived cells were detected on the 3rd, 7th, and 14th day after transplantation (Figures 7(a)–7(d)). In vivo differentiation of CEDPs was induced 3 days after transplantation shown by MLC2v expression on the 3rd, 7th, and 14th day after transplantation (Figure 7(a)).

The numbers of Isl1^+^ cells that survived 7 days after transplantation were quantified in dissected LVs. For this purpose, a 4 mm area was sectioned (400 sections, 10 *μ*m each). Six different planes were chosen (at 1.4 mm, 1.8 mm, 2.4 mm, 2.8 mm, 3.4 mm, and 3.7 mm) and ten sections around each plane were stained with anti-Isl1. Isl1^+^ cells were found between 1.4 and 3.7 mm (280 sections, approximately 2.8 mm) and counted. Based on Isl1^+^ cells counted on two nonsequential sections/planes (Supplementary Table S2) we produce a piecewise object of interpolating splines and plot this expression. Then we evaluate the total number of cells by taking the integral of the function in the interval [110,370] and found that Isl1^+^ cells were approximately 1,31 × 10^5^ (Figure 7(e)). Considering that Isl1^+^ cells percentage is ~60% of CEDPs (Figure 3(c)); surviving Isl1^+^ cells represent 21.8% of the initially injected.

## 4. Discussion

Genetic strategies based on tissue-specific activity of promoters are established to isolate ES-derived cell types. Cardiac specific promoters *α*-MHC and MLC2v were used to isolate highly purified cardiomyocytes during differentiation of genetically modified pluripotent ESCs [31–36]. In addition, the activity of developmental stage-specific promoters like Flk1, Isl1, and Nkx2.5 was used to isolate cardiac precursor cells from ES or iPS cells [21, 37–43].

### 4.1. Main Findings

We provide evidence that a novel population can be isolated by Pvec activity during ESCs differentiation. The endothelial specific activity of the (−2486, +24) fragment of mouse VE-cadherin promoter has been demonstrated in transgenic animals and during ESCs differentiation [13]. It has been further utilized for endothelial lineage tracing, endothelial specific knock-out mutant mice generation, and isolation of ES-derived endothelial cells [14, 16]. However, transient activation of Pvec was also found in hemogenic endothelium, a progenitor population that differentiated to endothelial and definitive hemopoietic cells lineages [19]. This demonstrates that precise timely evaluation of Pvec activity is crucial for selection experiments.

We have previously studied in detail the Pvec activity pattern during ESCs differentiation. Perhaps surprisingly, we found that it was transiently activated in a subset of Isl1^+^ multipotent cardiovascular progenitors between days 4 and 5; based on this finding, we selected cells surviving at this time-window. This genetically based approach resulted in isolation of Pvec^+^ cell population with endothelial- and cardiac-progenitor phenotypes but not endodermal or neuroectodermal phenotypes, an indication of specific selection. In our tissue culture system, utilizing activation of Wnt/*β*-catenin signaling resulted in propagation of the Pvec^+^ selected progenitor cells. Genetic approaches utilized Pvec to isolate endothelial cells. In these cases Pvec activity combined with FACS selection at day 8 of ES differentiation was used resulting in predominantly endothelial cell populations cells [14, 44]. Isolated CEDPs on the other hand is a complex cell population that has the ability for self-renewal for at least 30 days and is comprised from endothelial, cardiac, endocardial, and mesenchymal cell types. Therefore, it clearly represents a different and novel cell population. The most striking difference between our study and others resulting in cardiac progenitor cells (CPCs) isolation is that we chose a strategy allowing simultaneous isolation and expansion of endothelial and cardiac progenitors. For this purpose, our strategy was based on two steps: initially Pvec^+^ cells selection and subsequently Isl1^+^ cells expansion. Wnt/*β*-catenin induced expansion of Isl1^+^ cells was in agreement with previous reports [45]. However, quite unexpected self-renewal driven by Wnt/*β*-catenin pathway activation was observed for endothelial progenitors as well, a finding that requires further investigation. Thus, two independent populations with self-renew capacity were isolated, in contrast to previous studies, where endothelial cells were derived only after CPCs differentiation [21, 41]. Endothelial progenitors could improve their differentiation and viability properties of cardiac progenitors in a synergistic manner.

### 4.2. Wnt Signaling

Wnt signaling is pivotal for progenitor cells proliferation in a variety of tissues, such as the skeletal muscle [46] and the neuronal [47, 48], hemopoietic [49], and cardiac [50, 51] tissues. Its role during cardiac differentiation has been studied in detail and its activation was found to be essential for proper cardiac specification, progenitor expansion, and myocardial growth. Specifically, activation of Wnt/*β*-catenin at the emergence of Isl1^+^ cells resulted in the clonal expansion of these cardiovascular progenitors [45, 50]. At a later stage, when Wnt/*β*-catenin is downregulated, Isl1^+^ cells differentiated to cardiac and endothelial cells [21].

We report that, in addition to cardiac, proliferation of endothelial cells was achieved under our culture conditions in Pvec^+^ selected cells. This finding is important, given the inconclusive results of previous studies on the role of Wnt signaling on endothelial progenitors [52]. Whether positive regulation of endothelial progenitors by GSK3 inhibition is the result of Wnt activation on a subset of Pvec^+^ cells or, alternatively, whether they derive from Isl1^+^ cardiovascular progenitors remains to be seen.

### 4.3. Self-Renewal of Cellular Populations

Isolation of progenitor cells with cardiovascular potential, able to self-renew, is particularly interesting, since their differentiation is restricted to cardiac, endothelial, and smooth muscle cell types. These cells could provide a source for cardiac regeneration upon transplantation [39, 53, 54]. In the present study, we used the VE-cadherin promoter activity pattern to isolate a novel, dual-progenitor cell population. These cells could self-renew, under Wnt/*β*-catenin pathway stimulation and differentiate further to endothelial, cardiac, and smooth muscle cells. Dual differentiation to endothelial cells and cardiomyocytes can be viewed as an advantage, since it results in formation of vascularized transplants, with enhanced survival potential after transplantation [55].

### 4.4. Cell-Survival In Vivo

Survival of mouse origin transplanted cells in rat recipient animals was based on Isl1 expression as a positive marker. The Isl1^+^ cell population declines during mammalian embryogenesis and can be detected predominantly in sinoatrial node in the adult heart but not in the left ventricle [56]. Weinberger et al. reported Isl1^+^ cells presence in the sinoatrial node of the adult heart, a subpopulation derived from embryonic Isl1^+^ cells. In our experiments, we described the isolation of the Isl1^+^ population that emerges during cardiovascular development and differentiates to form a major part of the adult heart (left ventricle, part of atria, and the outflow tract endothelium). Future studies will show its differentiation potential to specific cardiac subpopulations.

In our study, we transplanted CEDPs in the left ventricle and all immunocytochemical and PCR analysis was performed in isolated left ventricle portion of the heart. Therefore, we believe that Isl1^+^ cells represent a genuine CEDPs-derived population. Based on this feature, we showed that transplanted cells survive and differentiate in the adult heart for at least 14 days, which underlines their potential in cell-based therapy for myocardial infarct.

The concept of a novel cardiac and endothelial population described in this manuscript could be potentially valuable for the field of cardiac regeneration when followed in the human system with necessary modifications. The strategy for clinical exploitation would require establishment of culture conditions of human ESCs (or iPSCs) towards cardiac differentiation in defined, serum-free medium. The VE-cadherin^+^/Isl1^+^ cell population can then be isolated and used further based on early VE-cadherin expression between days 4 and 5 in human by FACS sorting [57].

## 5. Conclusions

We isolated and characterized a novel cardiovascular progenitor cell population that has the capacity to self-renew and further differentiate to endothelial, cardiac, and smooth muscle cells in vitro and in vivo. These cells can be used in a cell-based therapy for myocardial infarct and in drug screening. Further characterization is needed, focusing on myocardial structures, such as the T-tubule system, the sarcolemma complex, and gap junctions.

## Supplementary Material

Pvec- and Pvec-EGFP-genetically modified ESC clones were characterized in respect to pluripotency and differentiation properties (Supplementary Figure S1). ES clone A11 morphology, survival and differentiation was examined during hugromycin selection (Supplementary Figure S2). Endothelial maturation stage of Pvec^+^ cells grown in the presence of SB-216763 was assessed by vWF and CD39 expression (Supplementary Figure S3). Pvec^+^ cells did not differentiate in the presence of SB-216763 (Supplementary Figure S4). Emergence of SMA^+^ and vimentin^+^ mesenchymal-origin cells after 12 days of CEDPs differentiation (Supplementary Figure S5). Control experiments demonstrating absence of neuroectodermal and endodermal markers expression at d10 of CEDPs differentiation (Supplementary Figure S6). Morphology of spheres formed by CEDPs embedded in alginate hydrogel for 5 days (Supplementary Figure S7). RT-PCR analysis demonstrating absence of pluripotency markers Oct3/4 and Nanog expression in the heart of transplanted animals (Supplementary Figure S8). Sequence alignment data supporting species-selectivity of the MLC2v primer set used to identify putative mouse-derived ventricular myocytes in the rat heart (Supplementary Figure S9). Supplementary Table S1 shows the sequences of all PCR primers used in the present study. Quantification of Isl1^+^ cells in cryosections of CEDPs-injected rat hearts by immunocytochemistry (Supplementary Table S2).
