# Supplementary material for: Isolation of an ES-Derived Cardiovascular Multipotent Cell Population Based on VE-Cadherin Promoter Activity
Source: Stem Cells Int. 2016 Dec 22;2016:8305624. doi: 10.1155/2016/8305624 (PMC5215608; doi:10.1155/2016/8305624)
Supplement: Supplementary file 1 — Pvec- and Pvec-EGFP-genetically modified ESC clones were characterized in respect to pluripotency and differentiation properties (Supplementary Figure S1). ES clone A11 morphology, survival and differentiation was examined during hugromycin selection (Supplementary Figure S2). Endothelial maturation stage of Pvec+ cells grown in the presence of SB-216763 was assessed by vWF and CD39 expression (Supplementary Figure S3). Pvec+ cells did not differentiate in the presence of SB-216763 (Supplementary Figure S4). Emergence of SMA+ and vimentin+ mesenchymal-origin cells after 12 days of CEDPs differentiation (Supplementary Figure S5). Control experiments demonstrating absence of neuroectodermal and endodermal markers expression at d10 of CEDPs differentiation (Supplementary Figure S6). Morphology of spheres formed by CEDPs embedded in alginate hydrogel for 5 days (Supplementary Figure S7). RT-PCR analysis demonstrating absence of pluripotency markers Oct3/4 and Nanog expression in the heart of transplanted animals (Supplementary Figure S8). Sequence alignment data supporting species-selectivity of the MLC2v primer set used to identify putative mouse-derived ventricular myocytes in the rat heart (Supplementary Figure S9). Supplementary Table S1 shows the sequences of all PCR primers used in the present study. Quantification of Isl1+ cells in cryosections of CEDPs-injected rat hearts by immunocytochemistry (Supplementary Table S2). [file 8305624.f1.pdf]

Supplementary Table 1 is presenting the PCR primer list used.

**Gene name**	**forward primer 5->3**	**reverse primer 5->3**	**product size cDNA**
VE-Cadherin	GTAACCCTGTAGGGAAAGAGTCCATT	GCATGCTCCCGATTAAACTGCCCATA	260bp
PECAM-1	GTCATGGCCATGGTCGAGTA	CTCCTCGGCGATCTTGCTGAA	260bp
Flk1	TTTGGCAAATACAACCCTTCAGA	GCAGAAGATACTGTCACCACC	133bp
Nfatc1	AGGTGCAGCCCAAGTCTCAC	GTGGCCATCTGGAGCCTTCT	620bp
Nrg1	CCAATGGCCACATTGCCAATAGGT	AGCCTGGCCTGTAATTCTTCCTGT	229bp
Isl-1	GCAGCTCCAGCAGCAGCAACCCA	TGGGAGCTGCGAGGACATCGATGC	253bp
Nkx2.5	AGCCGCCCCCACATTTTACCCG	GCGAGAAGAGCACGCGTGGCT	178bp
Gata4	GCGGAAGGAGGGGATTCAAA	TGAATGTCTGGGACATGGAGC	230bp
cTnT	AGCCCACATGCCTGCTTAAA	TCTCGGCTCTCCCTCTGAAC	115bp
MLC2v	ACTTCACCGTGTTCCTCACGATGT	TCCGTGGGTAATGATGTGGACCAA	254bp

MLC2a	AAGGGAAGGGTCCCATCAACTTCA	AACAGTTGCTCTACCTCAGCAGGA	202bp
AFP	CTTCCCTCATCCTCCTGCTAC	ACAAACTGGGTAAAGGTGATGG	234bp
FOXA2	CCGTGAAGATGGAAGGGCAC	TCATTCCAGCGCCCACATAG	210bp
Pdx1	CCTTTCCCGAATGGAACCGAG	GGTCAAGTTCAACATCACTGCC	264bp
Nestin	CATACAGGACTCTGCTGGAGG	AGGTGCTGGTCCTCTGGTAT	130bp
Tubb3	CTTTTCGTCTCTAGCCGCGT	TCCCAGAACTTGGCCCCTAT	94bp
Pax6	TGAGAAGTGTGGGAACCAGC	CACTCCGCTGTGACTGTTCT	214bp
NANOG	CCAGTGGAGTATCCCAGCAT	GTTGGTCCAGGTCTGGTTGT	159bp
SOX2	AAAGGGTTCTTGCTGGGTTT	AAACAAGACCACGAAAACGG	156bp
b-actin	GTGACGTTGACATCCGTAAAG	GCCGGACTCATCGTACTC	244bp
gapdh	AGGTCGGTGTGAACGGATTTG	GGGGTCGTTGATGGCAACA	94bp

Supplementary Figure S1.

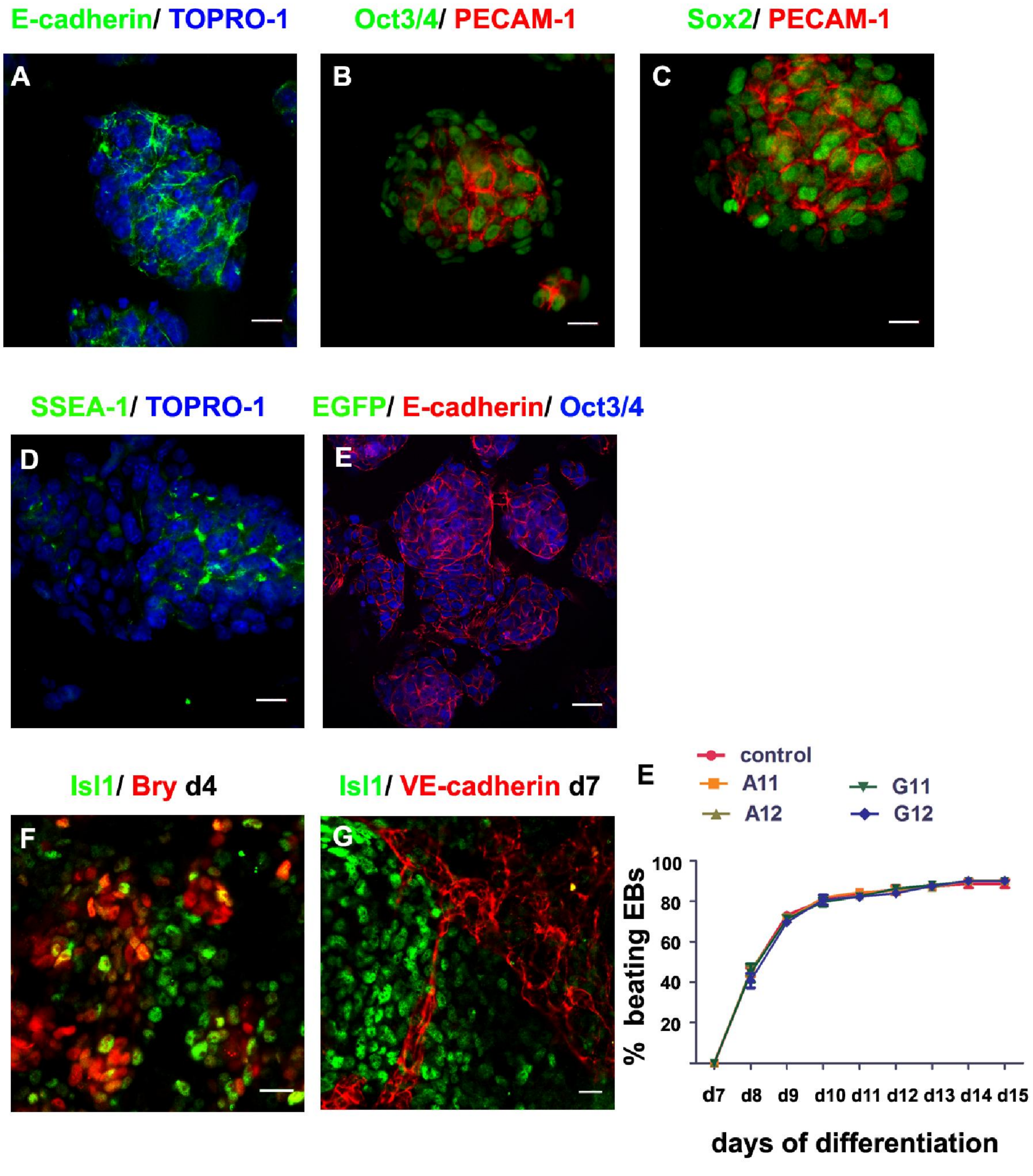

Supplementary Figure S1: Characterization of Pvec- and Pvec-EGFP- genetically modified ESCs. (A-E) Undifferentiated cells from clone A11 and G11 express pluripotency markers Oct3/4, Sox2, PECAM-1, SSEA-1 and E-cadherin but not EGFP, as shown by immunostaining.

DNA was stained with TOPRO-1. (F) Mesodermal Bry^{+} cells and Isl1^{+} cardiovascular progenitor cells in A11 derived-EBs at d4. (G) Endothelial VE-cadherin^{+} cells were typically located adjacent to Isl1+ progenitor cells in A11 derived-EBs at d7. (H) Statistical analysis of beating activity in clones A11, A12, G11, G12 and control (E14T ESCs) during differentiation days 7-15 (an EB was considered as beating if contained one or more beating areas). ***P <0,001 vs control. Data were evaluated by Repeated-measures two-way ANOVA, followed by Bonferroni post-hoc analysis for multiple comparisons. Probability values P <0.05 were considered significant. Scale bars: 20 μm

Supplementary Figure S2.

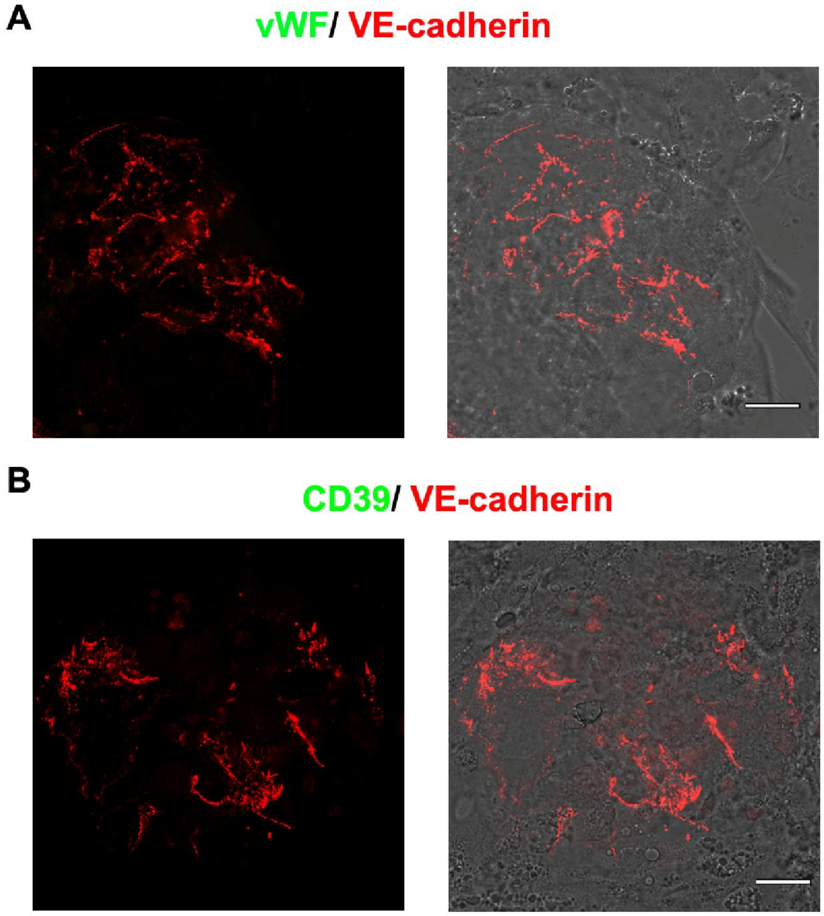

Supplementary Figure S2: Pvec+ cells express VE-cadherin but not vWF or CD39 after double immunostaining with anti-VE-cadherin and anti- vWF (A) or anti- CD39 (B). Right panels are the immunofluorescence images superimposed on the background of phase-contrast optics. Scale bars: (A-B) 20 μm

Supplementary Figure S3.

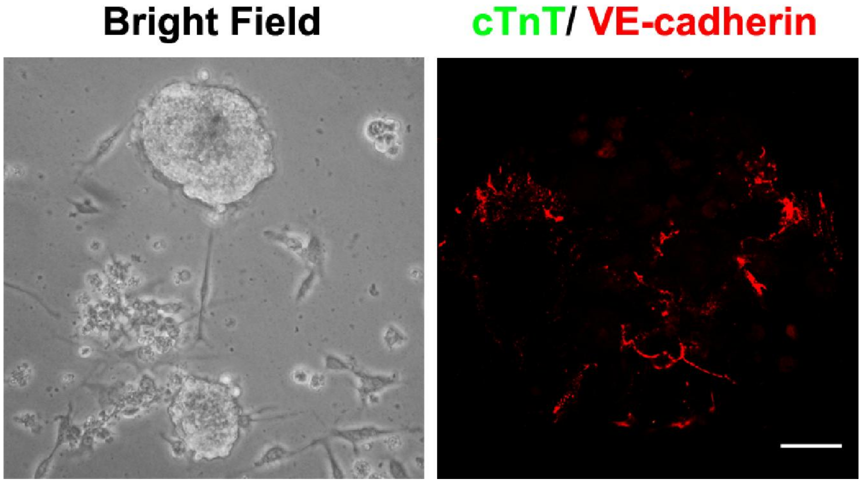

Supplementary Figure S3: (A) Typical image of spheres formed in the presence of SB-216763 after 5 days of CEDPs differentiation. (B) cTnT^{+} cells did not form under these conditions after 10 days of CEDPs differentiation, after immunostaining with anti-cTnT and anti-VE-cadherin. Scale bar: (B) 20 μm

Supplemental video of a beating area after 15 days of CEDPs differentiation.

Supplementary Figure S4.

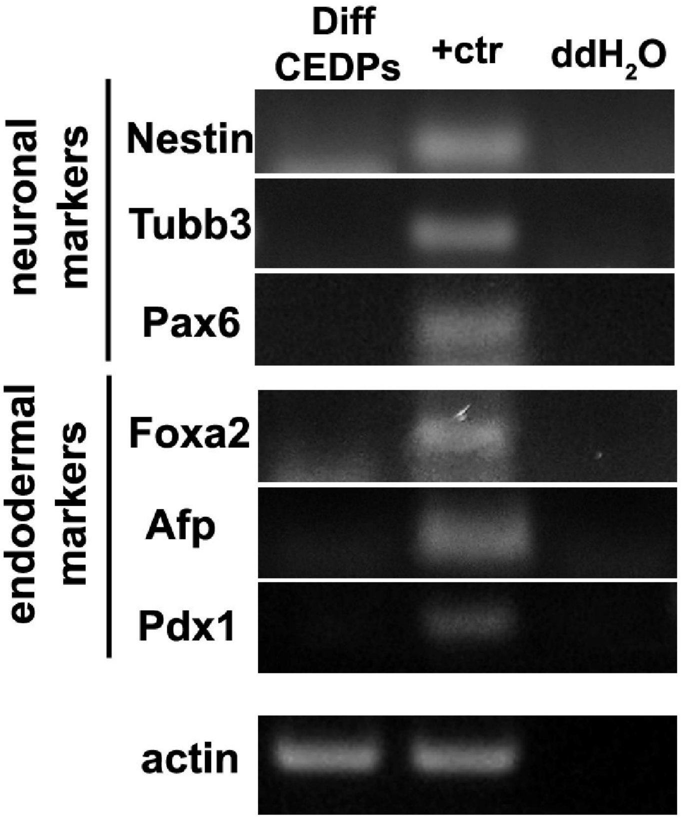

Supplementary Figure S4: RT-PCR analysis of CEDPs differentiation products at d10 showed absence of neuroectodermal and endodermal markers.

Supplementary Figure S5.

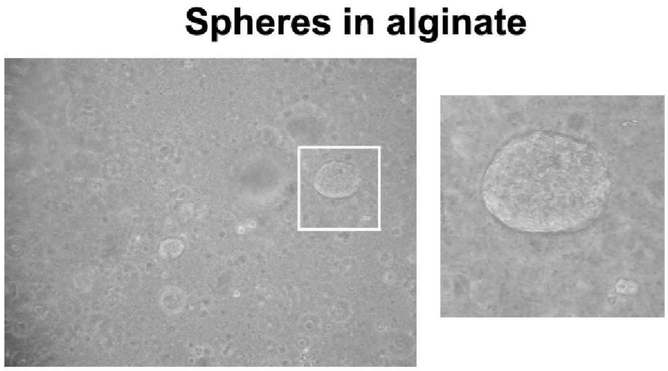

Supplementary Figure S5: Optical microscope image of spheres formed in gelatinised alginate hydrogel at day 5 of CEDPs differentiation (magnification corresponds to marked area).

Supplementary Figure S6.

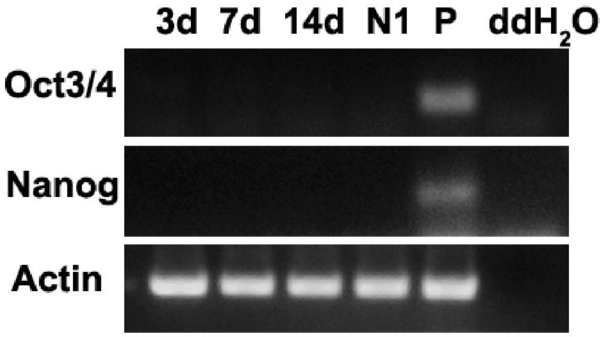

Supplementary Figure S6: Absence of pluripotency markers Oct3/4 and Nanog in transplanted animals by RT-PCR analysis.

Supplementary Figure S7.

Download GenBank Graphics

Mus musculus myosin, light polypeptide 2, regulatory, cardiac, slow (Myl2), mRNA

Sequence ID: ref|NM_010861.3| Length: 633 Number of Matches: 1

Range 1: 280 to 303 GenBank Graphics ▼ Next Match ▲ Previous Match

Score	Expect	Identities	Gaps	Strand
48.1 bits(24)	1e-04	24/24(100%)	0/24(0%)	Plus/Plus

```
Query  1    ACTTCACCGTGTTCCTCACGATGT  24
            ||||||||||||||||||||||||
Sbjct  280  ACTTCACCGTGTTCCTCACGATGT  303
```

Download GenBank Graphics

Mus musculus myosin, light polypeptide 2, regulatory, cardiac, slow (Myl2), mRNA

Sequence ID: ref|NM_010861.3| Length: 633 Number of Matches: 1

Range 1: 510 to 533 GenBank Graphics ▼ Next Match ▲ Previous Match

Score	Expect	Identities	Gaps	Strand
48.1 bits(24)	1e-04	24/24(100%)	0/24(0%)	Plus/Minus

```
Query  1    TCCGTGGGTAATGATGTGGACCAA  24
            ||||||||||||||||||||||||
Sbjct  533  TCCGTGGGTAATGATGTGGACCAA  510
```

Download GenBank Graphics

Rattus norvegicus myosin light chain 2 (Myl2), mRNA

Sequence ID: ref|NM_001035252.2| Length: 677 Number of Matches: 1

See 1 more title(s)

Range 1: 262 to 285 GenBank Graphics Next Match Previous Match

Score	Expect	Identities	Gaps	Strand
32.2 bits(16)	0.84	22/24(92%)	0/24(0%)	Plus/Plus

Download GenBank Graphics

Rattus norvegicus myosin light chain 2 (Myl2), mRNA

Sequence ID: ref|NM_001035252.2| Length: 677 Number of Matches: 1

See 1 more title(s)

Range 1: 492 to 515 GenBank Graphics Next Match Previous Match

Score	Expect	Identities	Gaps	Strand
32.2 bits(16)	0.84	22/24(92%)	0/24(0%)	Plus/Minus

```
Query  1    TCCGTGGGTAATGATGTGGACCAA  24
            ||||||||| |||||||| |||||
Sbjct  515  TCCGTGGGTGATGATGTGAACCAA  492
```

Supplementary Figure S7: Sequence alignment data supporting species-selectivity of the MLC2v primer set used to identify putative mouse-derived ventricular myocytes in the rat heart.

Supplementary Table S2 is presenting the mean of $Isl1^{+}$ cells counted in two non-sequential sections.

sampled sections #	$Isl1^{+}$ number of cells counted # mean
110 (108 and 112)	65
140 (138 and 142)	472
190 (188 and 192)	736
240 (238 and 242)	755
280 (278 and 282)	435
330 (328 and 332)	356
370 (368 and 372)	71
